# Increase in Alveolar Septal Width Is a Histological Predictor of Chronic Lung Allograft Dysfunction and Survival in Lung Transplant Recipients—A Longitudinal Study

**DOI:** 10.3390/jcm14186368

**Published:** 2025-09-09

**Authors:** Stefan Kuhnert, Anna M. Rotert, Janine Sommerlad, Athiththan Yogeswaran, Martin Reichert, Ingolf Askevold, Andreas Hecker, Christian Koch, Andreas Bräuninger, Stefan Gattenlöhner, Werner Seeger, Matthias Hecker, Peter Dorfmüller

**Affiliations:** 1 Department of Internal Medicine II, University Hospital Giessen and Marburg, Justus-Liebig-University Giessen, Klinikstrasse 33, 35392 Giessen, Germany; stefan.kuhnert@innere.med.uni-giessen.de (S.K.); janine.sommerlad@innere.med.uni-giessen.de (J.S.); athiththan.yogeswaran@innere.med.uni-giessen.de (A.Y.); werner.seeger@innere.med.uni-giessen.de (W.S.); 2 Department of General, Visceral, Thoracic and Transplant Surgery, University Hospital Giessen, Justus-Liebig-University Giessen, Rudolf-Buchheim-Strasse 6, 35392 Giessen, Germany; anna.m.rotert@chiru.med.uni-giessen.de (A.M.R.); martin.reichert@chiru.med.uni-giessen.de (M.R.); ingolf.askevold@chiru.med.uni-giessen.de (I.A.); andreas.hecker@chiru.med.uni-giessen.de (A.H.); 3 Department of Internal Medicine, Universities of Giessen and Marburg Lung Center (UGMLC), Institute for Lung Health (ILH), Cardio-Pulmonary Institute (CPI), 35392 Giessen, Germany; peter.dorfmuller@patho.med.uni-giessen.de; 4 Department of Anesthesiology, Intensive Care Medicine and Pain Medicine, University Hospital Giessen and Marburg, Justus-Liebig-University, 35392 Giessen, Germany; christian.koch@chiru.med.uni-giessen.de; 5 Department of Pathology, University of Giessen, Justus-Liebig University, 35392 Giessen, Germany; andreas.braeuninger@patho.med.uni-giessen.de (A.B.); stefan.gattenloehner@patho.med.uni-giessen.de (S.G.)

**Keywords:** lung transplantation, histological prediction of CLAD, alveolar septal width, survival

## Abstract

**Background:** Chronic lung allograft dysfunction (CLAD) occurs in up to 50% of patients within the first five years after lung transplantation (LuTX) and represents the main complication and cause of death regarding this surgery. Alveolar septal widening in transbronchial biopsies has shown an association with acute humoral allograft rejection. We aimed to explore histological markers that could predict the development of CLAD before its clinical manifestation. **Methods:** We retrospectively analyzed transbronchial biopsies taken at three time points from 57 patients who underwent LuTX between February 2010 and July 2019, 26 of whom developed CLAD up to November 2022. The biopsies were analyzed by microscopic morphometry and quantitative reverse transcription PCR to identify predictors of CLAD. **Results:** CLAD development was associated with increased alveolar septal width (ASW) as early as the first year post-LuTX (5.46 ± 0.76 µm versus 4.59 ± 0.44 µm; *p* < 0.001). The ASW in later biopsy timepoints predicted survival in multivariate models (last timepoint: hazard ratio 1.885, 95% confidence interval 1.086–3.269). Collagen (COL1A1 and COL3A1) expression was significantly increased in samples from patients who developed CLAD compared with those who did not. The increase in ASW was paralleled by interstitial deposition of COL1A1 and COL3A1 and a decrease in both the carbon monoxide (DLCO) diffusing capacity of the lung and the DLCO/alveolar volume. **Conclusions:** We report a new histologic approach for early assessment of risk of CLAD in patients who have undergone LuTX. The ASW represents a pre-symptomatic, continuous, and widely distributed change within the lung parenchyma that is accessible to transbronchial biopsy.

## 1. Introduction

Lung transplantation (LuTX) offers an ultimate treatment option for patients with end-stage lung diseases. Despite improvements in donor selection, surgical techniques, and post-operative care, including immunosuppression and graft-related disease management, the median survival after LuTX is only 6.7 years [1]. Chronic lung allograft dysfunction (CLAD) occurs in 50% of patients within the first five years after LuTX and represents the main cause of death after the first year of LuTX [1,2]. CLAD manifests predominantly as bronchiolitis obliterans syndrome (BOS) [3], while less common manifestations are restrictive allograft syndrome (RAS), mixed phenotypes, and undefined phenotypes [1]. Post-transplantation surveillance includes spirometric measurements of lung function, imaging techniques, collection of bronchoalveolar lavage fluid, and histological examination of transbronchial lung biopsies (TBBs) [4]. TBBs are performed to detect episodes of acute rejection (including acute cellular rejection [ACR], lymphocytic bronchiolitis [LB], and antibody-mediated rejection [AMR]), which represent a strong risk factor for the development of CLAD [5,6,7,8,9]. ACR and LB are defined by the circumferential perivascular infiltration and diffuse or circumferential bronchiolar submucosal infiltration of mononuclear cells, respectively, and are quantitatively and qualitatively graded [10,11,12]. The histological diagnosis of AMR is challenging, considering its less specific pathological characteristics, such as capillary inflammation or endothelitis [13,14]. Recently, Calabrese and co-workers suggested diffuse alveolar septal widening in TBBs as a potential “alert signal” and histomorphological surrogate marker for AMR [15].

CLAD may be caused by recurrent acute rejections, which could be a source of repeated inflammatory episodes; we hypothesized that a continuous increase in alveolar septal width (ASW) might predict CLAD and be associated with a decline in lung function and prognosis. We also investigated whether differences in allograft ASW in the first year after transplantation predict CLAD. Furthermore, we aimed to identify fibrosis-related biomolecular markers that could be responsible for a continuous increase in ASW.

## 2. Methods

### 2.1. Patients

A retrospective cohort analysis was conducted using a register of lung transplant recipients at the University of Giessen Lung Center (UGLC). Fifty-seven patients who underwent LuTX in two collaborating LuTX centers (UGLC and Kerckhoff Clinic Bad Nauheim) between February 2010 and July 2019 were selected. Of these, 26 patients had confirmed CLAD and known timepoint of CLAD onset, and 31 patients had no decline in baseline lung function up to November 2022. The survival status (registered as all-cause mortality) of every participant was known to this date (Figure S1). The follow-up time did not differ significantly between the groups. None of the patients had been diagnosed with SARS-CoV-2 infection before their final sampling timepoint. Patients received low-dose macrolides early after LuTX as standard primary prophylaxis against CLAD, unless intolerable side effects occurred. Maintenance immunosuppressive therapy included tacrolimus, mycophenolate, and corticosteroids. Induction immunosuppressive therapy was not administered. Clinically or spirometrically significant non-minimal acute cellular rejection [10] was treated with augmented immunosuppression, most commonly with a pulse of high-dose corticosteroids. A detailed description of LuTX candidate selection, surgical and interventional techniques, and post-LuTX management is given in the Supplementary Materials. This study was approved by the institutional research ethics board (AZ 135/21).

### 2.2. Surveillance of Lung Transplant Recipients

After LuTX, lung function tests were performed weekly during the hospital stay and inpatient rehabilitation, approximately once per month post-discharge for the first half of the postoperative year, and at least once every 3 months thereafter. Every lung function test consisted of spirometry combined with body plethysmography and carbon monoxide diffusion measurement. Body plethysmography (Master Screen Body, Jaeger, Wuerzburg, Germany) and blood gas analysis were performed according to the American Thoracic Society and European Respiratory Society Guidelines [16]. All patients were encouraged to use a home spirometry device daily. High-resolution computed tomography (CT) of the chest with inspiratory and expiratory imaging was performed yearly. Bronchoalveolar lavage and TBBs were routinely performed every three months for the first postoperative year, and yearly thereafter for three to five years as well as when clinically indicated. BOS was treated with montelukast; extracorporeal photopheresis was used if BOS was progressing rapidly. In case of suspected AMR, plasmapheresis was applied.

### 2.3. Definition of CLAD

The definition of, diagnostic criteria for, and phenotyping of CLAD followed the ISHLT consensus report on Chronic Lung Allograft Dysfunction [3]. CLAD is defined as a decline in forced expiratory volume in 1 s (FEV_1_) by ≥20% from baseline for at least 3 months, after extensive diagnostic workup (including CT and endoscopic pulmonary sampling) to exclude treatable causes or complications [3].

The baseline FEV_1_ value was computed as the mean of the best two postoperative FEV_1_ measurements (taken >3 weeks apart) in the first 12 months after LuTX. Baseline values of forced vital capacity (FVC) and total lung capacity (TLC) were defined as the mean of the two measures at the time of the two best FEV_1_ values.

### 2.4. CLAD Phenotypes

For all phenotypes, a persistent decline in FEV_1_ by ≥20% from baseline was the starting point for further subtyping. Spirometric signs of obstruction (FEV_1_/FVC < 0.7) with preserved TLC and exclusion of opacities in chest CT led to a diagnosis of BOS. Restrictive ventilatory impairment (TLC decline ≥10% from baseline) with CT opacities and without signs of obstruction was diagnosed as RAS. Cases of simultaneous obstructive and restrictive pattern were termed mixed phenotypes if persistent CT opacities were also present; otherwise, they were classed as undefined phenotype [3] (Figure S2, Table S1).

### 2.5. Sampled Histological Material

Samples were taken at different timepoints (TP1 and TP2) after transplantation. TP1 samples were taken within the first year after transplantation (before development of CLAD); TP2 samples were taken later, either as last sample before or first sample after the onset of CLAD (Figure S1). For correlation analysis with diffusion capacity, additional samples without defined timepoint were taken. Samples graded AX^10^ and samples with inadequately assessable alveolar parenchyma were excluded. We prioritized samples of good quality. It is important to note that our measurement of alveolar septal width (ASW) reflects the overall thickness of the alveolar septum, which includes interstitial connective tissue, capillaries, and supporting structures. This is distinct from the ultrastructural air–blood barrier (ABB), whose mean thickness in physiological conditions is approximately 1 µm. Thus, ASW values of 4–7 µm observed in both CLAD and non-CLAD samples fall within the possible range for septal thickness and should not be equated with direct ABB measurements.

### 2.6. Morphometric Analysis

**Figure 1:**
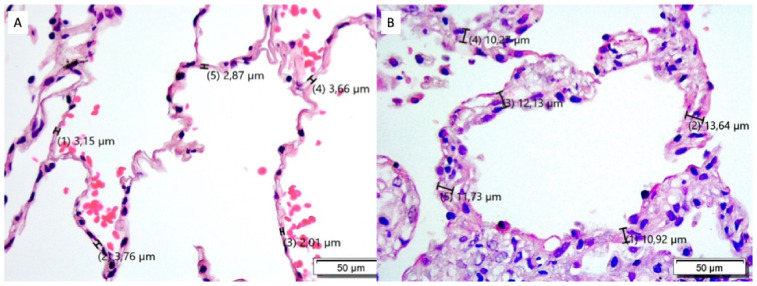
Examples of hematoxylin- and eosin-stained slides at ×40 magnification. (**A**) Transbronchial biopsy of a control patient. (**B**) Transbronchial biopsy of a patient who developed chronic lung allograft dysfunction.

Quantitative morphometry was performed by two pathologists independently at ×40 magnification in hematoxylin- and eosin-stained slides (Figure 1) using an Olympus BX 41TF light-microscope (Olympus, Tokyo, Japan), Olympus SC50 camera (Olympus, Tokyo, Japan), and CellSens Entry 2.3 software (Evident Europe GmbH, Hamburg, Germany). For each sample, 85 manual measurements of ASW were taken (5–25 measurements per field in ≥5 fields per slide), with exclusion of areas with intraseptal inflammatory cells or fibroblasts with visible nuclei to avoid artificial increases in ASW. For validation, manual ASW measurements were compared with septum area measured using two standardized methods. In the first method, septum area (including intraseptal inflammatory cells and fibroblasts with visible nuclei) was measured within a region of interest (ROI) with a constant area of 325.25 µm^2^. This method was applied to five separate alveolar septa per field in 30 randomly chosen TBBs from the groups with and without CLAD. In the second method, the total septal tissue area within a ×40 magnified field with adequately assessable alveolar architecture (including intraseptal inflammatory cells or fibroblasts with visible nuclei, but excluding pre-/post-capillary microvessels, extraseptal detritus, and adherent/intra-alveolar cells) was expressed relative to the number of displayed alveoli. This method was applied to TP2 samples of five randomly chosen patients with and without CLAD (5 fields per slide). ASW including intraseptal cells was measured manually in the same fields (50 measurements per field) for comparison with the second standardized method. Mean values of the manual measurements of both observers were determined. In case of substantial inter-observer discrepancy, the slide was reviewed together (both observers), discussed, and re-assessed. Discrepancies were mainly due to intraseptal cells, such as inflammatory cells, artificially increasing ASW (Figure S3).

Once this analytic and methodologic groundwork was achieved and validated, we sought to simplify our method for practical purposes and hence for applicable routine hospital conditions. To this end, we simplified our measurements and reduced the number of microscopic fields from the initial 5 fields to 2 fields at a magnification of ×200 (objective ×20). We took 10 measurements per field in samples at TP1. In parallel, we used the same 2 microscopic fields for an artificial intelligence (AI)-supported analysis with the neuronal network-based solution from Evident, TrueAI^®^ (Evident Europe GmbH, Hamburg, Germany). We limited the number of patients to 10 patients who would develop CLAD and 10 from the non-CLAD group for this approach, to see if statistical significance could be reached at lower numbers. We chose the 10 best preserved slides regarding tissue and section quality in HE staining for both groups.

### 2.7. Molecular Analysis

For molecular analysis, we chose biopsies of TP1 and TP2 of 10 patients with CLAD and 10 patients without CLAD, respectively. Areas with alveolar parenchyma only, excluding larger blood-vessels and airways, were marked under microscopic control to obtain RNA of corresponding areas in formalin-fixed, paraffin-embedded blocks using manual macrodissection. RNA was extracted and purified using the Maxwell Rapid Sample Concentrator RNA FFPE Kit (Promega, Walldorf, Germany) and reverse transcribed using the Archer FusionPlex kit (ArcherDX Inc., Boulder, CO, USA). Quantitative PCR was performed using StepOne RealTime PCR (Thermo Fisher Scientific, Waltham, MA, USA) with TaqMan assays for collagen 1A1 (COL1A1), COL3A1, COL4A1, COL5A1, cathepsin B, and the housekeeping gene ribosomal protein lateral stalk subunit P0 (RPLP0). All RealTime PCR analyses were performed as replicates for each probe to generate a mean cycle threshold (Ct) value. Genes for which Ct values were >33 and experiments with differences >1 for replicates were excluded. RNA expression relative to RPLP0 is described as ΔCt.

### 2.8. Statistical Analysis

Adherence to Gaussian distribution was determined using the Kolmogorov–Smirnov test and visual assessment of histograms. Normally distributed variables are displayed as mean ± standard deviation and compared using bidirectional *t*-tests; non-normally distributed variables are displayed as median [Q1–Q3] and compared using non-parametric Mann–Whitney U tests; and categorical variables are displayed as *n* (%) and compared using Chi-square tests. Changes in ASW over time were analyzed by a general linear model and compared between groups using repeated measures ANCOVA with covariates (recipient age and sex). A linear logistic regression model was used to determine the association between selected relevant donor- and recipient-derived results, as well as periprocedural variables, and early ASW.

Associations with mortality were assessed in univariate Cox regression analysis including selected relevant donor and recipient characteristics and peri-operative parameters (Table 1 and Table S2). Recipient age and sex and all variables that showed a significant association with mortality were included in a multivariate, stepwise, backward Cox regression model. The ASWs at TP1 and TP2 were investigated individually due to strong multicollinearity in multivariate models. Missing data were not imputed. Mortality prediction was assessed by receiver operating characteristic analysis with Youden’s index. Survival and the combined endpoint CLAD-free survival were assessed using Kaplan–Meier analyses with log-rank tests.

For all analyses, *p* < 0.05 was considered significant. Analyses were performed using the statistical software R 3.5 (the R Foundation, Vienna, Austria) and SPSS 26.0 (IBM, Armonk, NY, USA). For moderation analysis of CLAD at TP2, the PROCESS module for SPSS was used [17].

## 3. Results

### 3.1. Cohort

The donor and patient characteristics were widely comparable between the groups with and without subsequent development of CLAD. Overall donor smoking status also did not show significant differences, with the exception of donors with a history of strong smoking, who were more abundant in the CLAD group (Table 1 and Table S2). In the CLAD group, 15 patients (58%) had BOS, 1 patient (4%) had RAS, 3 patients (12%) had mixed phenotypes, and 7 patients (27%) had an undefined phenotype (Figure S2). The CLAD phenotypes did not show significant differences in their ASW at TP1 or TP2.

### 3.2. Sampled Histological Material

TP1 (*n* = 57) and TP2 (*n* = 57) samples were taken 10–378 days and 361–3194 days after LuTX, respectively. For correlation analysis, 42 additional samples were taken at undefined timepoints. The time intervals from LuTX to TP1 and TP2 were not significantly different between the groups with and without CLAD (*p* = 0.987 and *p* = 0.39, respectively) (Figure S4).

### 3.3. ASW Validation

The manual ASW measurements correlated strongly with standardized measurement of the alveolar septal area relative to a constant ROI (R^2^ = 0.9134) and the ratio of total alveolar septal area to the number of visible alveoli (R^2^ = 0.8449) (Figure S5). The ratio of total septal area to number of alveoli was significantly greater in patients who developed CLAD than in those who did not (2661.10 ± 623.84 µm^2^ versus 1531.70 ± 309.86 µm^2^; *p* < 0.001). Manual measurements of ASW (including intraseptal cells) in the same fields showed a similar pattern (7.56 ± 1.32 µm versus 4.93 ± 0.62 µm; *p* < 0.0001).

### 3.4. ASW and CLAD

**Figure 2:**
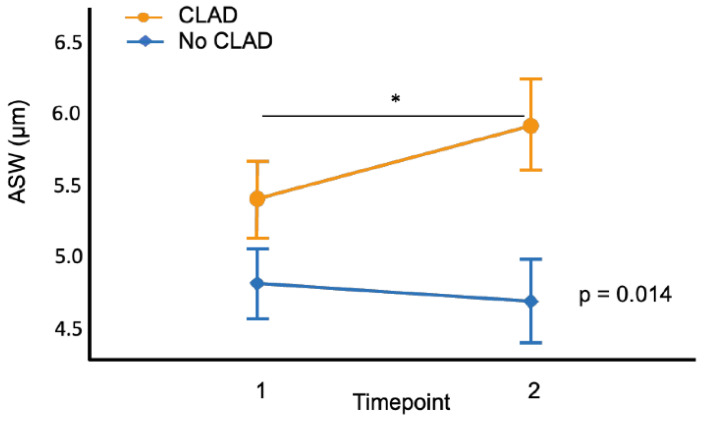
ASW over time in patients who subsequently developed CLAD and those who did not. ASW, alveolar septal width; CLAD, chronic lung allograft dysfunction. * *p* < 0.05.

The ASW was significantly higher in patients who developed CLAD than in those who did not, at both timepoints (TP1: 5.46 ± 0.76 µm versus 4.59 ± 0.44 µm; TP2: 6.07 ± 0.57 µm versus 4.37 ± 0.55 µm; all *p* < 0.001). In receiver operating characteristic analysis, the ASW at TP1 (area under the curve (AUC) 0.723, 95% confidence interval (CI) 0.589–0.857, *p* = 0.004) was a significant predictor of CLAD (Figure S6). The ASW increased over time in patients who developed CLAD (TP1 < TP2, *p* = 0.005), while it remained stable in patients without CLAD. The trajectories of both groups differed significantly (*p* = 0.006; Figure 2).

### 3.5. ASW and Diffusing Capacity of the Lung for Carbon Monoxide (DLCO)

**Figure 3:**
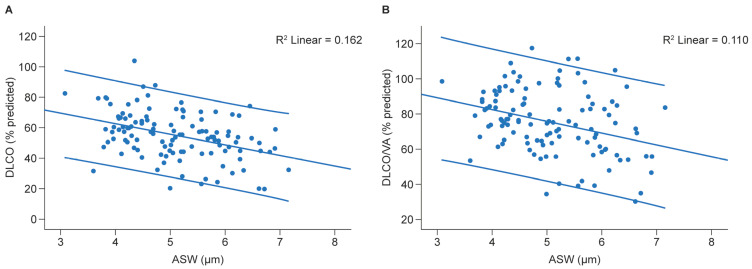
Scatter plots showing the association of (**A**) DLCO and (**B**) DLCO/VA with corresponding ASW measurements. Error bars indicate 95% confidence intervals. ASW, alveolar septal width; DLCO, diffusing capacity of the lung for carbon monoxide; VA, alveolar volume.

The alveolar septal thickening, total surface area, and diffusion gradient are the most relevant determinants of DLCO [18]. The association of DLCO with the development of CLAD has been shown recently [19]. In our cohort, the ASW showed a moderate correlation with the DLCO (percent predicted) (R^2^ = 0.162, *p* < 0.001) and a moderate correlation with DLCO/accessible alveolar volume (DLCO/VA) (R^2^ = 0.110, *p* < 0.001) (Figure 3).

### 3.6. Factors Associated with ASW

We investigated donor characteristics and perioperative factors for their association with early measurements of ASW at TP1. Linear regression analysis showed no significant associations with ASW at TP1 (Table 2). For later samples, at TP2, only the number of CMV reactivations and CMV status mismatch between the donor and recipient had a low but significant impact on ASW (regression coefficient 0.250, 95% CI 0.054–0.447, *p* = 0.013, R^2^ = 0.101 (Figure S7) and −0.43, 95% CI −0.748–0.112, *p* = 0.008, R^2^ = 0.04). Furthermore, there was no significant difference in the incidence of ACR and LB between the CLAD group and stable patients and no association of an increase in ASW with concurrent acute rejection. None of the patients had AMR.

### 3.7. Extracellular Molecular Markers for ASW

We selected samples for molecular pathology to investigate the contribution of extracellular matrix constituents to ASW.

After applying our exclusion criteria, only COL1A1, COL3A1, COL4A1, and cathepsin B yielded evaluable results. The CLAD group showed significantly higher expression of *COL1A1* (*p* = 0.03) and *COL3A1* (*p* = 0.01) than the group without CLAD, whereas *cathepsin B* (*p* = 0.67) and *COL4A1* (*p* = 0.10) showed no significant difference between the two groups (Figure S8).

We found a weak but significant positive correlation between the ASW and the expression levels of *COL1A1* (R^2^ = 0.1135, *p* = 0.036) and *COL3A1* (R^2^ = 0.1139, *p* = 0.033) (Figure S9).

### 3.8. Survival

Twenty patients (35%) died during the observation period (up to November 2022). In our univariate regression analysis, donor arterial oxygen pressure (PO_2_) under ventilation with 100% oxygen, the duration of invasive mechanical ventilation for the recipient post-LuTX, the length of stay (LOS) in the intensive care unit (ICU), the use of extracorporeal membrane oxygenation (ECMO) during surgery, the cumulative number of CMV reactivations up to TP2, and the ASW at TP1 significantly predicted mortality. (Table 3; see Table S3 for results of all univariate parameters). The LOS in the ICU was excluded from the multivariate models owing to multicollinearity with the duration of ventilation post-LuTX (R = 0.787). In the multivariate model, an independent association of the ASW at TP1 with mortality could not be confirmed.

**Figure 4:**
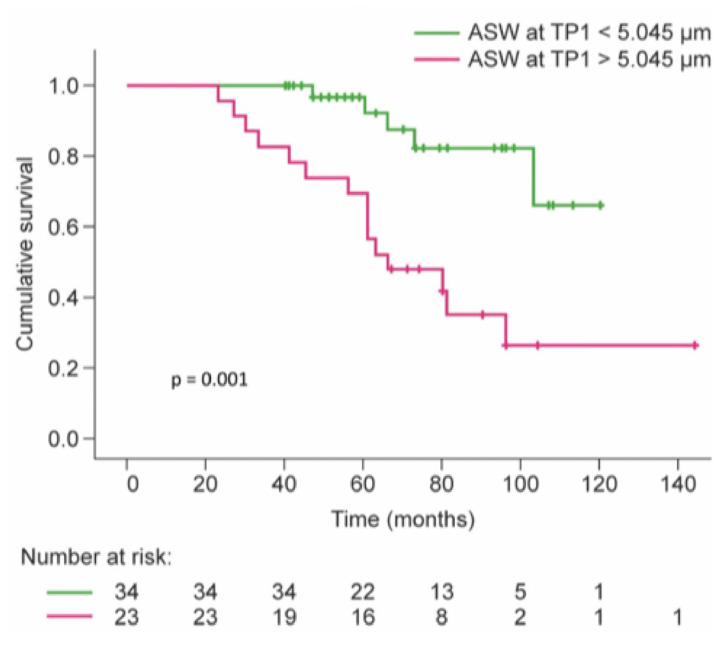
Kaplan–Meier survival curves for patients with ASW early after lung transplant (TP1) over and under 5.045 µm. ASW, alveolar septal width; TP, timepoint.

Dichotomizing the ASW at TP1 at 5.045 µm (based on Youden’s index), we observed a significantly reduced survival (*p* = 0.001, Figure 4) and CLAD-free survival (*p* < 0.001, Figure S10) compared with those with lower ASW. To investigate the impact of later samples, we also tested the ASW at TP2 for survival. The donor PO_2_ under ventilation with 100% oxygen, the use of ECMO during surgery, and the cumulative number of CMV reactivations were also significantly associated with mortality in the two multivariate models that included the ASW at TP2 (Table 3). Survival at TP2 was not moderated by the occurrence of CLAD up to this timepoint. Neither the CLAD status at TP2 nor its interaction with the ASW at TP2 was significant in this model.

### 3.9. ASW Measurement in Clinical Routine

**Figure 5:**
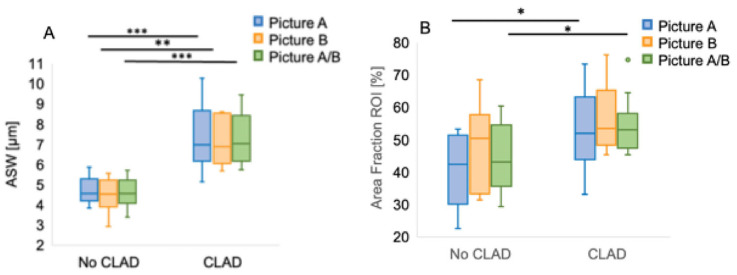
(**A**) Manual measured ASW and (**B**) AI-determined ROI area fractions. Each method took 10 measurements per field. Horizontal lines indicate the median, boxes indicate the interquartile range, and whiskers indicate the 95% confidence interval. ASW, alveolar septal width; CLAD, chronic lung allograft dysfunction; ROI, region of interest. * *p* < 0.05. ** *p* < 0.01, *** *p* < 0.001.

We developed a time-saving, clinically applicable method for determining ASW under routine hospital conditions by reducing the number of microscopic fields and measurements. This study compared 10 patients from each group (CLAD vs. non-CLAD), with manual measurements being performed in two histologic fields at 200× magnification. In Field A, the mean of 10 ASW measurements was significantly higher in the patients who later developed CLAD than in those who remained CLAD-free (7.38 ± 1.57 µm vs. 4.71 ± 0.64 µm; *p* < 0.001). Field B showed comparable results (7.12 ± 1.18 µm vs. 4.49 ± 0.87 µm; *p* = 0.002). Similarly, the mean of all measurements from both fields revealed significant differences between the groups (CLAD: 7.25 ± 1.28 µm vs. non-CLAD: 4.60 ± 0.73 µm; *p* < 0.001) (Figure 5A; Table S4).

We then applied an AI-based approach using automated measurements of ROI area fractions from the same histologic fields. In Field A, the patients who developed CLAD showed significantly higher values than those who did not (CLAD: 52.26 ± 12.77% vs. non-CLAD: 40.54 ± 11.80%; *p* = 0.047). In Field B, the difference was not statistically significant (CLAD: 56.93 ± 11.41% vs. non-CLAD: 48.31 ± 12.68%; *p* = 0.13). However, when combining measurements from both fields, the patients who developed CLAD again exhibited significantly higher mean values compared to the patients without CLAD (54.59 ± 9.08% vs. 44.42 ± 10.27%; *p* = 0.031) (Figure 5B; Table S4).

## 4. Discussion

Our study indicates a striking association of CLAD development with an early increase in ASW in the post-LuTX period. ASW measurement might represent a new tool for the early identification of high-risk patients who could be eligible for risk-adjusted graft-related disease management. The ASW in later stages is predictive for survival in multivariate models. By contrast, we did not find any association of increased ASW with the intra-individual incidence of acute rejection, and the latter was not a predictor of CLAD, which reflects the rather episodic and spatially heterogeneous occurrence of perivascular and bronchiolar inflammation in the natural history of CLAD.

In contrast with recent findings of Calabrese and co-workers [15], none of our patients were diagnosed with an episode of AMR at the time of sampling. The ASW values in our study were also generally lower than those reported by Calabrese and co-workers. This might be due to (i) a different pathomechanism, given the hyperacute nature of AMR-related graft rejection (e.g., interstitial edema vs. interstitial deposition of collagen through increased recruitment of fibroblasts), and/or (ii) technical differences in morphometric measurements (e.g., recording of ASW in areas without intraseptal inflammatory cells or fibroblasts with visible nuclei).

Multivariate Cox regression analysis suggested a worse outcome in patients with a higher AWS at TP2 without moderation by the occurrence of CLAD. Even in early measurements at TP1, ASW > 5.045 µm was associated with worse survival, although we were unable to confirm this in multivariate models.

We believe that higher ASW measurements shortly after transplant may raise concern about future lung function decline and promote early treatment. To the best of our knowledge there are no comparable analyses to date.

Morphological alterations associated with CLAD development include persistent activation of (myo-)fibroblasts and consecutive intrabronchial and parenchymal deposition of collagen as an endpoint of the pro-fibrotic cascade led by transforming growth factor (TGF)β expression [20,21,22]. We assumed that deposition of collagens 1A1, 3A1, 4A1, and 5A1, which are predominantly found in the extracellular matrix in the lungs of patients with idiopathic interstitial fibrosis (IPF)/usual interstitial pneumonia (UIP), could contribute to increased ASW [23,24,25]. Our biomolecular analysis indicates the association of greater ASW with higher expression of collagens 1A1 and 3A1, but not collagens 4A1 and 5A1. These collagens that appear to be involved in the increase in ASW are ubiquitarily expressed at the protein level and immunohistochemical detection did not convince us: stainings with COL4, for example, appear almost as a histochemical staining of the interstitium, like staining with Masson’s trichrome or Elastica von Gieson, and are strongly positive in the whole interstitial space. COL1A1 is, to the contrary, only faintly visible in IHC stainings of CLAD lungs, although it is nicely expressed in fibroblastic foci of UIP lungs, for example. In order to discriminate CLAD from non-CLAD in immunohistochemistry, these antigens do not appear suitable, at least not for discrimination in a dichotomic way of expressed vs. not expressed.

Morrone and colleagues recently showed that cathepsin B is involved in the TGFβ-driven pro-fibrotic cascade and is overexpressed in the bronchoalveolar lavage fluid and parenchyma of allografts with BOS [26]. We found a similar distribution of cathepsin B expression in samples from patients who developed CLAD and those from stable patients. This could indicate latent pro-fibrotic activity in all allografts, but to a varying degree, which limits the usefulness of cathepsin B in discriminating stable patients from those at risk of developing CLAD.

Nonetheless, as in the IPF/UIP ratio, an increase in the ASW due (in part) to deposition of interstitial collagen represents a morphologically perceivable alteration of the alveolo–capillary interface that is reflected in the decline of the DLCO and DLCO/VA. We found a moderate to strong negative correlation between the DLCO (and DLCO/VA) and ASW. Recently, declines in the DLCO were reported to be independently associated with CLAD development [19]. Additionally, a low baseline DLCO was correlated with poor allograft survival [19]. These findings strongly support our results identifying increased ASW as a predictor of CLAD and post-LuTX survival. Gas diffusion across the alveolar–capillary interface is fundamentally governed by Fick’s law: the rate of gas transfer is directly proportional to the surface area and concentration (or partial pressure) gradient, and inversely proportional to the thickness of the air–blood barrier (ABB) [27]. Our observed correlation between ASW measurements and the DLCO aligns with this principle: patients with CLAD with thicker septa show reduced diffusing capacity. However, testing of the DLCO uses carbon monoxide (CO) as the test gas, which is highly soluble and binds hemoglobin with great affinity—making it diffusion-limited and sensitive to barrier abnormalities. In contrast, oxygen (O_2_) is less soluble and more likely to be perfusion-limited under normal conditions but becomes diffusion-limited earlier when the barrier is thickened (e.g., in interstitial edema or fibrosis) [28]. Thus, impairment of O_2_ transfer could be even more pronounced than suggested by DLCO reductions, a clinically relevant consideration, since subtle diffusion defects may already compromise oxygenation even when the DLCO is only modestly reduced. This also highlights a key limitation of conventional pulmonary function testing: FEV_1_ and even the DLCO may fail to detect early interstitial remodeling. Incorporating sensitive tools for early ABB injury, such as ASW morphometry, could therefore improve early CLAD detection and patient management.

We found the cumulative number of serological CMV reactivations and a mismatch of the CMV serostatus between the donor and recipient to be predictors of a higher ASW in TP2. Since CMV reactivation [29] and mismatch [30] is a known and well described factor for the development of CLAD, this finding clearly needs to be addressed in future studies.

### 4.1. Strengths and Limitations

Our study has several strengths. To the best of our knowledge, this is the first study to examine the association of ASW with the occurrence of CLAD and post-LuTX survival using routine clinical material. We used the updated definition of CLAD and modern histomorphometric analysis techniques. However, we were able to show that significant differences can be detected using a shortened protocol of manual measurement within a single histological field. Furthermore, we demonstrated that training an AI for this task appears to be feasible.

Our second validation method demonstrated that CLAD and non-CLAD cases can be distinguished using two approaches: first, by measuring the septal area in a single high-power microscopic field relative to the number of alveoli, using RGB-based morphometric software (CellSens Entry 2.3 software (Evident Europe GmbH, Hamburg, Germany)); second, by performing 50 manual ASW measurements within the same field. While RGB-based automated measurements are not yet standard in routine pathology labs (despite being widely available), conducting manual measurements using calibrated microscopes—such as those used to assess surgical margins in cancer diagnostics—is an established practice and could feasibly be applied for CLAD risk assessment.

The strength of our study is clearly not the identification of a molecular surrogate marker for the identification of an increased risk to develop CLAD. However, since transbronchial biopsies are not expected to be replaced as the gold standard diagnostic tool in post-LuTX care, we have identified ASW as a surrogate marker, as it as a morphologically assessable criterion that is accessible to a pathology lab with standard microscope equipment and standard microscope software that allows measurements on a micrometer scale.

For exploratory reasons, we sought to identify possible molecular correlatives for the increased thickness of alveolar septa: here, our results do not deliver an uncontested molecular candidate, but rather an indication that increased ASW in CLAD is due to increased deposition of specific collagens, such as COL1A1 and COL3A1, which are in fact ubiquitously expressed collagens that are difficult to use as specific surrogate markers.

Two principal processes may contribute to the increased ASW we observe in pre-CLAD biopsies: (i) interstitial edema due to perturbations in the lung water balance and (ii) progressive fibrotic collagen deposition. Post-transplant lungs are particularly prone to fluid imbalance because lymphatic drainage is interrupted, the vascular permeability is increased, and fluid shifts occur during reperfusion. Both donor and recipient conditions influence this vulnerability: donor lungs from extended criteria donors (e.g., older age, prior lung injury, or prolonged mechanical ventilation) are less efficient at fluid clearance, while recipient factors such as hemodynamic instability, use of ECMO, and perioperative fluid loading increase the likelihood of pulmonary edema [31]. Importantly, the earliest manifestation of such perturbations is interstitial edema, which is visible as widening of the alveolar septa. Thus, perioperative fluid management can emerge as a decisive determinant of the ASW measured in biopsies. However, our qPCR findings showing upregulation of COL1A1 and COL3A1 in CLAD cases support fibrosis as a structural driver of septal thickening. Nonetheless, ASW elevation in the first weeks after LuTX may reflect a dynamic interplay that is initially driven by fluid shifts and later solidified by ECM remodeling.

Potential biomarkers of CLAD have been investigated in recent [32,33] papers. For example, the potential of donor-derived cell-free DNA (dd-cfDNA) as a biomarker for monitoring lung allograft health has been recently reported [34]. The authors found elevated dd-cfDNA levels in 20 patients with CLAD during stable clinical condition, preclinical CLAD, and established CLAD. However, in their paper, the authors themselves question the applicability of their findings due to dd-cfDNA’s variability and lack of specificity: elevated levels may indicate allograft injury, but other conditions such as infections could also increase dd-cfDNA. Our current findings of a true histologic correlation for CLAD risk, increased ASW, could put a combined approach into perspective, on a molecular and morphologic level.

We recognize that our study did not directly measure microvascular permeability—a critical element in interstitial edema development and ASW increase. Notably, a recent investigation by Hudock et al. employed human lungs in ex vivo lung perfusion (EVLP) to functionally assess vascular permeability as a proxy for endothelial barrier integrity and early graft injury [35]. Building on this approach, future LuTX studies could incorporate permeability measures—such as imaging-based leakage assays during EVLP, permeability–surface area product quantification, or tracer-based PET/MRI techniques—to better characterize capillary–alveolar fluid exchange. Integrating such assessments into pre- and post-transplant surveillance may enhance early detection of microvascular compromise and provide valuable insights into the pathogenesis of interstitial remodeling leading to CLAD.

FEV_1_, while it is the current gold standard for CLAD diagnosis, primarily reflects obstruction of the larger airways and is relatively insensitive to early small airway or interstitial abnormalities. This limitation likely explains why structural changes such as increased ASW precede measurable declines in spirometry. High-frequency oscillometry (HFO) has recently emerged as a promising complementary tool, offering greater sensitivity to subtle alterations in peripheral airway mechanics and changes in the lung water balance. Incorporating HFO alongside morphometric markers such as the ASW could therefore improve early risk stratification in LuTX recipients.

During the study period, there were no changes in our surgical technique, post-LuTX management, system of data documentation and collection, intensive care treatment, immunosuppressive regimen, surveillance bronchoscopy sampling, or sample preservation technique. However, our study is limited by its retrospective and single-center design and the small sample size. Multicenter studies are warranted to confirm our results and evaluate the potential of this new parameter for routine assessment post-LuTX. We identify few factors underlying the observed increased ASW. Previously described risk factors [3] for the development of CLAD were not found to be relevant for the ASW in our cohort, except for the cumulative number of CMV reactivations and mismatch in the CMV status between the donor and recipient. Although none of the patients were suffering from severe CMV pneumonia, we included a variety of factors concerning donors, recipients, and the peri-operative period, but since these aspects were not the main focus of our work, the list is certainly incomplete.

### 4.2. Conclusions

We report a new histologic approach for early assessment of risk of CLAD in patients who have undergone LuTX. We have identified ASW > 5.045 µm as an individual predictor of survival, and increases in ASW are paralleled by interstitial deposition of the collagens 1A1 and 3A1 and a decrease in the DLCO and DLCO/VA. In contrast to the grading of ACR and LB, which is currently of clinical importance for the individual adjustment of immunosuppressive therapy, the ASW may represent a more continuous change in the lung parenchyma of patients at risk for developing CLAD and might be therapeutically addressed in the future. Multicenter studies are warranted to confirm our results and evaluate the potential of this new parameter for routine assessment post-LuTX.

## Figures and Tables

**Table 1:** Donor and recipient characteristics and peri- and post-operative factors.

		CLAD			
		No (*n* = 31)	Yes (*n* = 26)	All (*n* = 57)	*p*
Donor characteristics					
Age, years		52.9 ± 18.2	57.2 ± 16.4	55.0 ± 17.3	1.0
Sex, *n* (%)					0.94
	female	14 (45)	12 (48)	26 (46)	
	male	17 (55)	14 (54)	31 (54)	
Smoking history, *n* (%)					
	None	22 (71)	15 (58)	37 (65)	0.2
	Slight	3 (10)	3 (12)	6 (11)	0.84
	Medium	3 (10)	1 (4)	4 (7)	0.38
	Strong	0 (0)	5 (19)	5 (9)	0.01
	Missing	3 (10)	2 (8)	5 (9)	
Time on ventilation, days		4 [2–6]	3 [2–5]	3 [2–6]	0.58
PO_2_ 100%, mm Hg		452.0 ± 56.7	441.4 ± 69.4	447.4 ± 63.4	0.51
Blood type, *n* (%)					
	0	13 (42)	6 (23)	19 (33)	0.13
	A	14 (45)	16 (62)	30 (53)	0.22
	B	3 (10)	3 (12)	6 (11)	0.82
	AB	1 (3)	1 (4)	2 (4)	0.9
CMV serostatus, *n* (%)					0.14
	Negative	13 (42)	16 (62)	29 (51)	
	Positive	18 (58)	10 (38)	28 (49)	
Recipient characteristics					
Age, years		54.0 [38.0–63.0]	56.5 [56.5–61.0]	56.0 [43.5–61.0]	1.0
Sex, *n* (%)					0.6
	Female	15 (48)	15 (60)	30 (53)	
	Male	16 (52)	11 (40)	27 (47)	
Body mass index, kg/m^2^		23.30 ± 3.32	25.26 ± 4.26	23.22 ± 3.98	0.1
Lung disease, *n* (%)					0.2
	COPD/emphysema	2 (6)	6 (23)	8 (14)	0.07
	Fibrosis—IPF	8 (26)	4 (15)	12 (21)	0.34
	Fibrosis—other	12 (39)	12 (46)	24 (42)	0.57
	Cystic fibrosis	6 (19)	2 (8)	8 (14)	0.21
	Lymphangioleiomyomatosis	2 (6)	0 (0)	2 (4)	0.19
	Pulmonary hypertension	1 (3)	2 (8)	3 (5)	0.45
CMV serostatus, *n* (%)					0.06
	Negative	12 (39)	4 (15)	16 (28)	
	Positive	18 (58)	20 (77)	38 (67)	
	Missing	1 (3)	2 (8)	3 (5)	
Last LAS ^a^		38.5 [36.0–56.6]	36.9 [34.3–44.2]	37.1 [35.6–51.9]	0.94
Peri- and post-operative factors					
ECMO during surgery, *n* (%)					0.97
	No	20 (65)	16 (62)	36 (63)	
	Yes	11 (35)	9 (35)	20 (35)	
	Missing	0 (0)	1 (4)	1 (2)	
Incision–suture time, min		357.7 ± 75.3	357.4 ± 85.2	357.6 ± 79.2	1.0
Ischemic time, min		360 [240–420]	300 [257–375]	323 [241–390]	0.64
Number of RBC units transfused		3 [2–6]	3 [0–4]	3 [2–6]	0.47
Post-LuTX time on ventilator, h		48 [12–360]	144 [12–772]	72 [12–468]	0.14
LOS in ICU, d		16 [10–42]	26 [14–54]	20 [12–42]	0.24
Number of acute rejections ^b^		2 [1–5]	3 [1–4.5]	3 [1–5]	0.96
Cumulative CMV reactivation, *n* (%) ^b^		0.29 ± (0.74)	0.38 ± (0.94)	0.46 ± 1.0	0.36
LuTX center, *n* (%)					0.82
	Kerckhoff Clinic Bad Nauheim	3 (10)	3 (12)	6 (11)	
	UGLC	28 (90)	23 (88)	51 (89)	
Immunsuppression 1, *n* (%)					0.27
	Tacrolimus	31 (100)	25 (96)	56 (98)	
	Cyclophosporine	0 (0)	1 (4)	1 (2)	
Immunsuppression 2, *n* (%)					
	Mycophenolate	31 (100)	26 (100)	57 (100)	-
	CMV donor/recipient mismatch	13 (48)	14 (51)	57(100)	0.27

CLAD, chronic lung allograft dysfunction; CMV, cytomegalovirus; COPD, chronic obstructive pulmonary disease; ECMO, extracorporeal membrane oxygenation; ICU, intensive care unit; IPF, idiopathic pulmonary fibrosis; LAS, lung allocation score; LOS, length of stay; LuTX, lung transplantation; PO_2_ 100%, arterial oxygen pressure in mm Hg under ventilation with 100% oxygen; RBC, red blood cell; UGLC, University of Giessen Lung Center. ^a^ The lung allocation policy based on both urgency and waiting time was replaced with a scheme based on the LAS in December 2011 (three patients were allocated in the pre-LAS era). The LAS is a numerical value used by the United Network for Organ Sharing to assign relative priority for distributing donated lungs for transplantation and ranges from 0 to 100, with a higher score indicating greater priority. ^b^ From LuTX to timepoint 3. Data are presented as mean ± SD, median (Q1–Q3), or *n* (%). Additional donor and recipient characteristics and peri- and post-operative factors are presented in Table S2.

**Table 2:** Association of donor characteristics and peri- and post-operative factors with ASW at TP1.

	ASW at TP1		ASW TP2	
	B Coefficient (95% CI)	*p*	B Coefficient (95% CI)	*p*
Donor characteristics				
Age	0.004 (−0.008–0.016)	0.482	0.004 (−0.014–0.018)	0.78
Sex	−0.233 (−0.638–0.172)	0.254	−0.156 (0.857–0.223)	0.45
Height	−0.015 (−0.035–0.006)	0.166	−0.019 (−0.03–0.00)	0.09
Slight smoking	−0.342 (−0.987–0.304)	0.292	−0.63 (−0.987–0.860)	0.891
Medium smoking	−0.145 (−0.917–0.627)	0.707	−0.95 (−1.199–1.009)	0.86
Strong smoking	0.499 (−0.200–1.198)	0.158	0.728 (−0.272–1.728)	0.150
TLC	−0.126 (−0.300–0.047)	0.151	−0.2 (−0.405–0.057)	0.14
Days on ventilation	0.006 (−0.059–0.071)	0.852	−0.001 (−0.087–0.087)	0.995
CRP	0.001 (−0.001–0.003)	0.294	−0.063 (−0.003–0.002)	0.662
PO_2_ 100%	0.000 (−0.003–0.004)	0.774	−0.003 (−0.004–0.004)	0.982
Blood type A ^a^	0.244 (−0.193–0.681)	0.268	0.57 (−0.2–1.15)	0.06
Blood type B ^a^	0.500 (−0.199–1.198)	0.157	0.59 (−0.345–1.527)	0.21
Blood type AB ^a^	−0.809 (−1.917–0.300)	0.149	−0.404 (−1.89–1.083)	0.59
Rhesus factor	–	–	0.162 (−0.25–1.031)	0.227
CMV serostatus	0.318 (−0.082–0.717)	0.117	0.024 (−0.496–0.593)	0.860
Recipient characteristics				
Last LAS ^b^	0.005 (−0.007–0.017)	0.416	−0.018 (0.897–−0.02)	0.655
EBV mismatch D/R	0.046 (−0.338–0.430)	0.811	0.061 (−0.695–1.057)	0.67
CMV mismatch D/R	0.115 (−0.080–0.310)	0.242	−0.43 (−0.748–−0.112)	0.008 ^c^
TLC difference D/R	−0.022 (−0.273–0.230)	0.864	−0.069 (−0.393–0.256)	0.67
Peri- and post-operative factors				
ECMO before LuTX	0.560 (−0.304–1.424)	0.199	0.132 (−0.634–1.815)	0.34
ECMO during surgery	0.256 (−0.169−0.680)	0.233	0.004 (−0.571–0.578)	0.98
Incision–suture time	0.002 (−0.001–0.005)	0.239	−0.093 (0.005–0.002)	0.52
Ischemic time	−0.002 (−0.004–0.001)	0.184	−0.124 (−0.005–0.002)	0.36
Duration of MAP <60 mm Hg	0.001 (−0.004–0.007)	0.602	−0.124 (−0.009–0.004)	0.40
Cumulative vasopressor dose	6.662 × 10^−5^ (0.000–0.000)	0.103	−0.048 (0.000–0.000)	0.74
Post-LuTX time on ventilator	0.000 (0.000–0.001)	0.293	0.21 (0.00–0.001)	0.12
LOS in ICU	0.006 (−0.003–0.014)	0.176	0.006 (0.00–0.013)	0.051
Cumulative no. of acute rejections	-		0.063 (−0.266–0.428)	0.64
Cumulative no. of EBV reactivations	-		0.082 (−0.248–0.411)	0.62
Cumulative no. of CMV reactivations	-		0.323 (0.063–0.584)	0.016 ^d^

ASW, alveolar septal width; CI, confidence interval; CMV, cytomegalovirus; CRP, C-reactive protein; D/R, donor/recipient; EBV, Eppstein–Barr virus; ECMO, extracorporeal membrane oxygenation; ICU, intensive care unit; LAS, Lung allocation score; LuTX, lung transplantation; LOS, length of stay; MAP, mean arterial pressure; PO_2_ 100%, arterial oxygen pressure in mm Hg under ventilation with 100% oxygen; TLC, total lung capacity; TP, timepoint. ^a^ Blood type is estimated with reference to blood type 0. ^b^ The lung allocation policy based on both urgency and waiting time was replaced with a scheme based on the LAS in December 2011 (three patients were allocated in the pre-LAS era). ^c^ R^2^ = 0.04, ^d^ R^2^ = 0.101.

**Table 3:** Associations with mortality in univariate and multivariate regression models.

	Univariate		Multivariate	
	HR (95% CI)	*p*	HR (95% CI)	*p*
PO_2_ 100%	1.007 (1.000–1.014)	0.044	1.010 (1.003–1.017)	0.003
Age	0.988 (0.958–1.020)	0.462	0.994 (0.954–1.036)	0.781
Sex	0.514 (0.196–1.347)	0.176	0.613 (0.201–1.868)	0.389
ECMO during surgery	2.804 (1.121–7.014)	0.028	7.711 (2.339–25.421)	<0.001
Post-LuTX time on ventilator	1.002 (1.001–1.003)	0.004	1.000 (0.999–1.002)	0.55
Cumulative no. of CMV reactivations	1.616 (1.128–2.315)	0.009	1.800 (1.133–2.858)	0.013
ASW at TP1	1.636 (1.081–2.476)	0.020	0.143 (0.566–1.153)	0.392
ASW at TP2	1.823 (1.132–2.935)	0.013	1.885 (1.086–3.269)	0.024
CLAD at TP2	1.265 (0.513–3.120)	0.610	0.987 (0.00–26.18 × 10^3^)	0.998

ASW, alveolar septal width; CI, confidence interval; CLAD, chronic lung allograft dysfunction. CMV, cytomegalovirus; ECMO, extracorporeal membrane oxygenation; HR, hazard ratio; LuTX, lung transplantation; PO_2_ 100%, arterial oxygen pressure in mm Hg under ventilation with 100% oxygen; TP, timepoint. All available covariates were included in the univariate analysis (see Table S3), while only variables with a significance level of below 0.05 and recipient age and sex were used for the multivariate regression models.

## Data Availability

The datasets used and/or analyzed during the current study are available from the corresponding author on reasonable request.

## Supplementary Materials

The following supporting information can be downloaded at: https://www.mdpi.com/article/10.3390/jcm14186368/s1, Figure S1. Timepoints of sampling for patients who developed CLAD and those without CLAD; Figure S2. Frequency of different CLAD phenotypes in the study cohort; Figure S3. Standardized measurements of ASW with (A) alveolar septal area in relation to a region of interrest (ROI) with a constant area of 325.25 µm^2^ and (B) the ratio of total alveolar septal area to the number of visible alveoli in a x40-magnified field; Figure S4. Timepoints of sampling for patients who developed CLAD and those who did not. Horizontal lines indicate the median, boxes indicate the interquartile range, and whiskers indicate the 95% confidence interval; Figure S5. Manual measurements of ASW in correlation with (A) standardized measurements of alveolar septal area in relation to a ROI with a constant area of 325.25 µm^2^ and (B) the ratio of total alveolar septal area to the number of visible alveoli in a x40-magnified field; Figure S6. Receiver operating characteristic curves for the prediction of CLAD by ASW in early measurements (TP1). Diagonal segments are produced by ties; Figure S7. Scatter plot showing the association of ASW in late measurements (TP3) with the cumulative number of CMV reactivations. Error bars indicate the 95% confidence interval; Figure S8. ΔC_t_ values of expression of COL1A1, COL3A1, COL4A1, and cathepsin B in patients who developed CLAD and those who did not. One ΔCt value was excluded as an outlier. Horizontal lines indicate the median, and whiskers the 95% confidence interval; Figure S9. Manual measurements of ASW in correlation with ΔC_t_ values for expression of (A) COL1A1 (*p* = 0.036) and (B) COL3A1 (*p* = 0.033). Correlations of ASW with ΔC_t_ values for COL4A1 and cathepsin B expression were not significant (COL4A1: R^2^ = 0.197, *p* = 0.097; cathepsin B: R^2^ = 0.035, *p* = 0.973). One ΔCt value was excluded as an outlier; Figure S10. Kaplan–Meier CLAD – free survival curves for patients with ASW early after lung transplant (TP1) over and under 5.045 µm; Table S1. Definition of CLAD Phenotypes Adapted from Verleden et al. [3]; Table S2. Additional Donor and Recipient Characteristics and Peri- and Post-Operative Factors; Table S3. Associations with Mortality in Univariate and Multivariate Regression Models; Table S4. Manual short protocol measurements and KI analysis at TP1. References [36,37,38] are cited in the Supplementary Materials.

## Abbreviations

AI	artificial intelligence
ABB	air–blood barrier
ASW	alveolar septal width
ACR	acute cellular rejection
AMR	antibody-mediated rejection
AUC	area under the curve
BOS	bronchiolitis obliterans syndrome
CI	confidence interval
CLAD	chronic lung allograft dysfunction
CMV	cytomegalovirus
COL	collagen
CT	computed tomography
DLCO	diffusing capacity of the lung for carbon monoxide
ECMO	extracorporeal membrane oxygenation
FEV_1_	forced expiratory volume in 1 s
FVC	forced vital capacity
ICU	intensive care unit
ISHLT	international society for heart and lung transplantation
LB	lymphocytic bronchiolitis
LOS	length of stay
LuTX	lung transplantation
PCR	polymerase chain reaction
PO_2_	arterial oxygen pressure
RAS	restrictive allograft syndrome
ROI	region of interest
RPLP0	ribosomal protein lateral stalk subunit P0
TBB	transbronchial biopsy
TGFβ	transforming growth factor β
TLC	total lung capacity
TP	timepoint
UGLC	University Giessen Lung center
UIP	usual interstitial pneumonia
VA	alveolar volume
