# Supplementary material for: Increase in Alveolar Septal Width Is a Histological Predictor of Chronic Lung Allograft Dysfunction and Survival in Lung Transplant Recipients—A Longitudinal Study
Source: J Clin Med. 2025 Sep 9;14(18):6368. doi: 10.3390/jcm14186368 (PMC12471240; doi:10.3390/jcm14186368)
Supplement: Supplementary file 1 [file jcm-14-06368-s001.zip › jcm-3760612-supplementary.pdf]

Increase of alveolar septal width is a histological predictor of chronic lung allograft dysfunction and survival in lung transplanted patients – a longitudinal study

Stefan Kuhnert, MD*, Anna M. Rotert, MD*, Janine Sommerlad, MD, Athiththan Yogeswaran, MD, Martin Reichert, MD, Ingolf Askevold, MD, Andreas Hecker, MD, Christian Koch MD, Andreas Bräuninger, PhD, Stefan Gattenlöhner, MD, Werner Seeger, MD, Matthias Hecker, MD, PhD‡, Peter Dorfmüller, MD, PhD‡

*Both authors equally contributed to this study.

‡Both authors equally contributed to this study.

Contents

Supplemental methods .... 3~~2~~

Lung transplantation .... 3~~2~~

Immunosuppressive and antibiotic regimens, and early anti-infective prophylaxis .... 3~~2~~

Management of post-transplant hypertension .... 4~~3~~

Bronchoscopy and sampling .... 4~~3~~

Supplemental Figures .... 6~~5~~

Supplemental Tables .... 15~~11~~

Supplemental references .... 25~~20~~

Supplemental methods

Lung transplantation

Lung donor assessment, matching, procurement, preservation, and implantation were performed according to routine protocols.[1, 2] Lung transplantation (LuTX) was performed almost exclusively by bilateral thoracotomy. Two patients underwent single lung transplantation. Extracorporeal membrane oxygenation (ECMO) support was used during LuTX in 35.7% of the cases, with the trends varying over time. Data on intraoperative parameters were extracted from the NarkoData® anesthesia information management system (IMESO-IT GmbH, Giessen, Germany); the parameters included incision–suture time, ECMO support, cumulative vasopressor dose and furosemide dose, and fluid balance. Incision–suture time (in min) was defined as the time period from skin incision to the last suture of the surgical procedure. Graft cold ischemic time (in min), defined as the time period between donor clamp time and antegrade graft reperfusion in the recipient, was calculated from the DSO (Deutsche Stiftung Organtransplantation) graft information sheet and the NarkoData® anesthesia information management system. The perioperative numbers of red blood cell units, pooled platelet units, and fresh frozen plasma units administered were documented.

Immunosuppressive and antibiotic regimens, and early anti-infective prophylaxis

The standard perioperative antibiotic regimen consisted of ceftriaxone/clindamycin or an individualized regimen according to perioperative tissue cultures in patients with cystic fibrosis.

Standardized postoperative care was provided to all patients admitted to the intensive care units (ICUs) of the Department of Anesthesiology and Department of Pulmonology and Critical

Care Medicine, University Hospital Giessen and Marburg, Giessen, Germany. Clinical information for the ICU stay was obtained from the ICUData® patient documentation medical system (IMESO-IT GmbH, Giessen, Germany). Patients received a standard triple immunosuppressive regimen consisting of corticosteroids (1.5 g methylprednisone in the first 24 h after surgery followed by prednisolone 0.5mg/kg/day tapering to 0.25mg/kg/day at three months, 0.15mg/kg/day at six months, and 0.075mg/kg/day at twelve months post-transplant), tacrolimus (8–10 µg/L in the first 6 months, and 5–6 µg/L thereafter) or cyclophosphamide 80–150 µg/mL, and mycophenolate (1–3 µg/mL). Subsequent immunosuppression was tailored to the patient's rejection history, infection, bone marrow suppression, and kidney function. Mycophenolate could be switched to everolimus if kidney function deteriorated 6 months after LuTX. Anti-infective prophylaxis consisted of sulfamethoxazole/trimethoprim thrice weekly, voriconazole, and ganciclovir tailored to kidney function.

Management of post-transplant hypertension

In the outpatient setting, calcium antagonists were administered as calcineurin-inhibitor sparing agents to all patients who were diagnosed with post-transplant hypertension. All the patients received angiotensin-converting enzyme inhibitors or angiotensin II receptor blockers as additional antihypertensive medication at the discretion of the treating physician. Lung allograft performance was monitored serially for each patient, at least monthly in the first year after LuTX and then every 1 to 3 months indefinitely thereafter, or if clinically indicated.

Bronchoscopy and sampling

The bronchoscopic sampling procedure was described previously.[3] Surveillance bronchoscopies with bronchoalveolar lavage (BAL) and transbronchial lung biopsies were

performed on the 1st, 3rd, 6th, 9th, and 12th month post-transplantation and yearly thereafter or if clinically indicated. Fiberoptic flexible bronchoscopy was performed on sedated and orotracheally intubated patients, with 5ml of xylocaine applied bronchially.

BAL was performed with 150 mL of sterile saline in eight aliquots. After instillation, liquid was recovered manually with a 20 mL syringe. Transbronchial biopsies were acquired under direct radiologic control in peripheral allograft regions, with an 2mm Alligator Jaw-Step Swing Jaw biopsy forceps At least five samples of suitable size and quality were collected from the lower and upper lobe. Samples were directly transferred to formalin and sent to the department of pathology for further processing.

Supplemental Figures

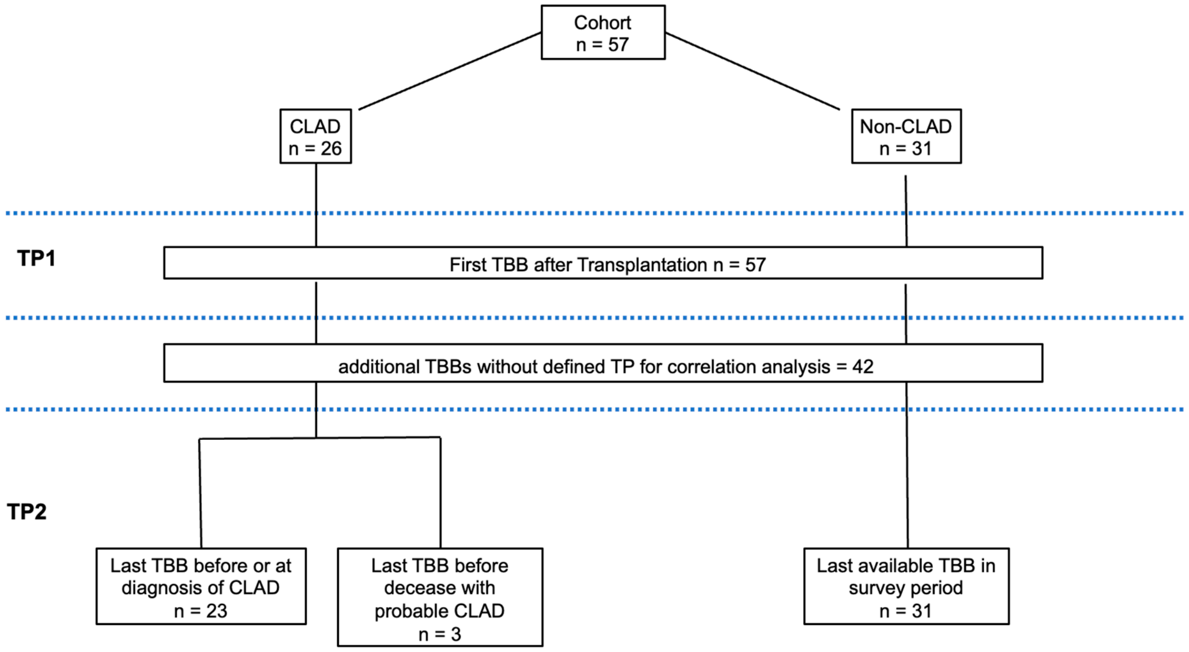

Figure S1 Timepoints of sampling for patients who developed CLAD and those without CLAD.

CLAD, chronic lung allograft dysfunction; TBB, transbronchial biopsy.

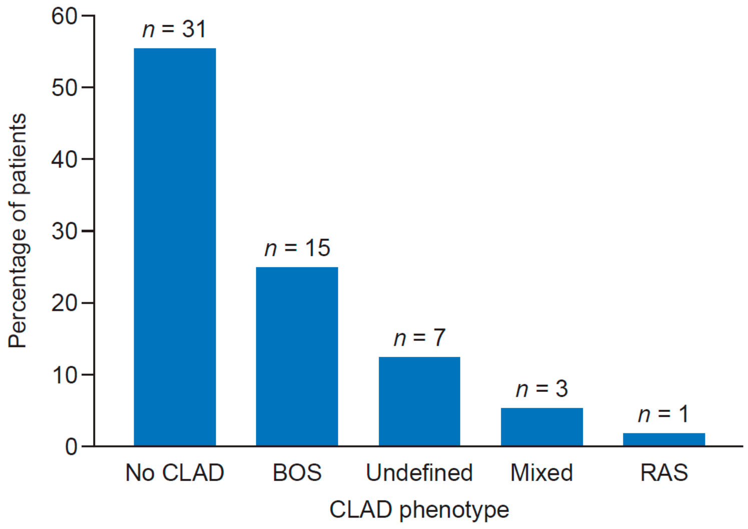

Figure S2 Frequency of different CLAD phenotypes in the study cohort. BOS, bronchiolitis obliterans syndrome; CLAD, chronic lung allograft dysfunction; RAS, restrictive allograft syndrome

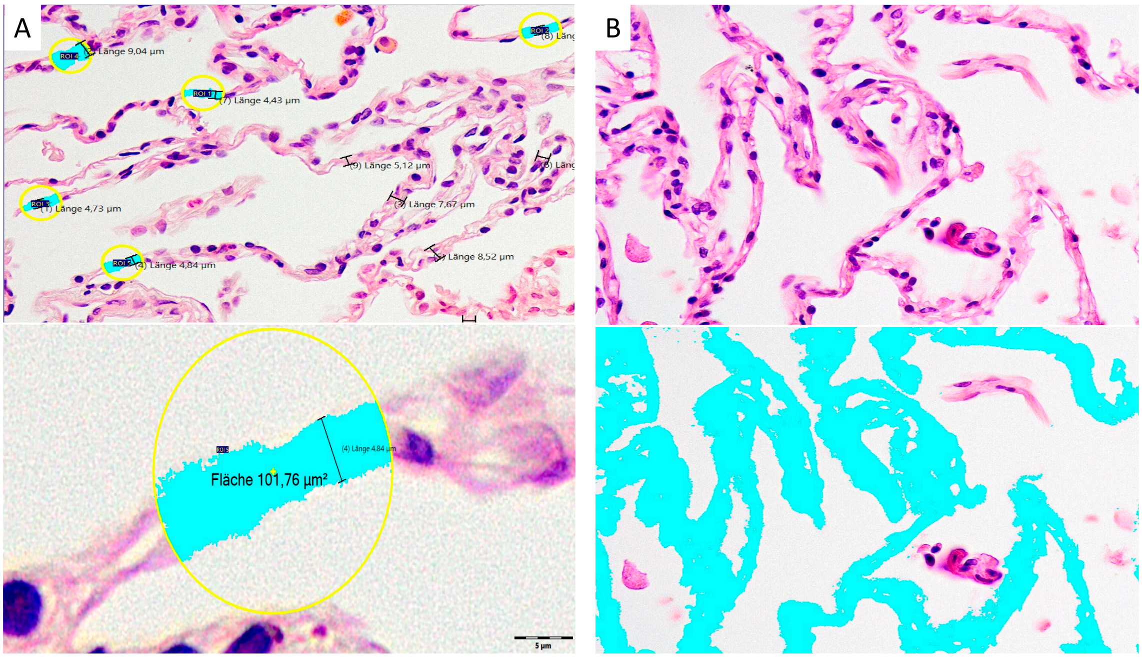

Figure S3 Standardized measurements of ASW with (A) alveolar septal area in relation to a region of interrest (ROI) with a constant area of 325.25 µm^2 and (B) the ratio of total alveolar septal area to the number of visible alveoli in a x40-magnified field.

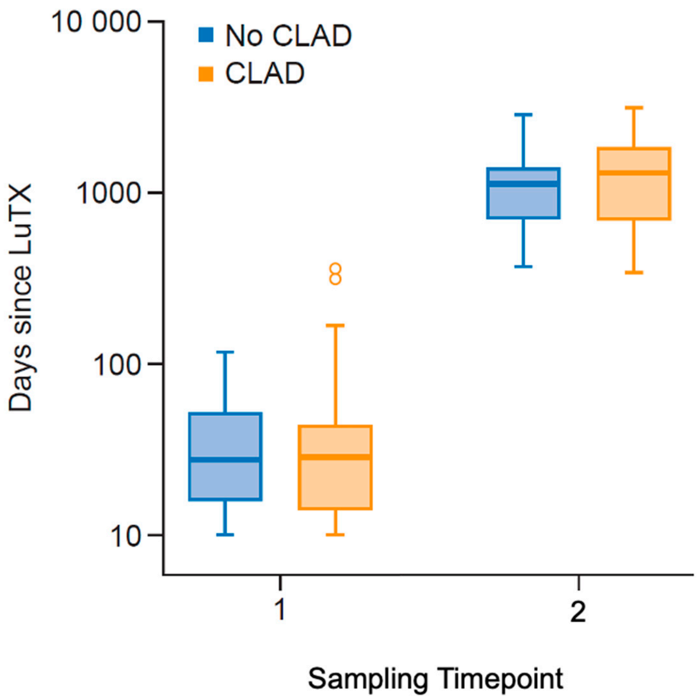

Figure S4 Timepoints of sampling for patients who developed CLAD and those who did not. Horizontal lines indicate the median, boxes indicate the interquartile range, and whiskers indicate the 95% confidence interval. CLAD, chronic lung allograft dysfunction; LuTX, lung transplantation.

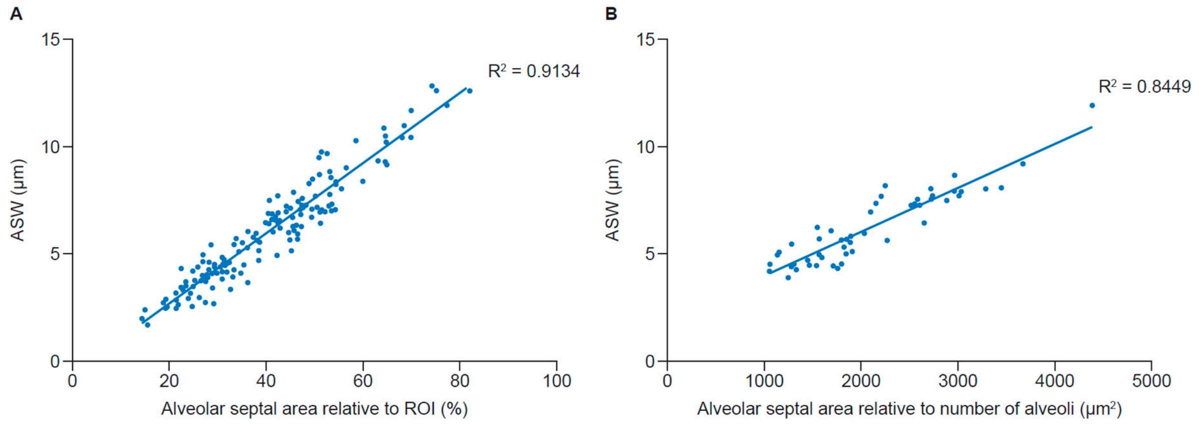

Figure S5 Manual measurements of ASW in correlation with (A) standardized measurements of alveolar septal area in relation to a ROI with a constant area of 325.25 μm^2 and (B) the ratio of total alveolar septal area to the number of visible alveoli in a x40-magnified field. ASW, alveolar septal width; ROI, region of interest.

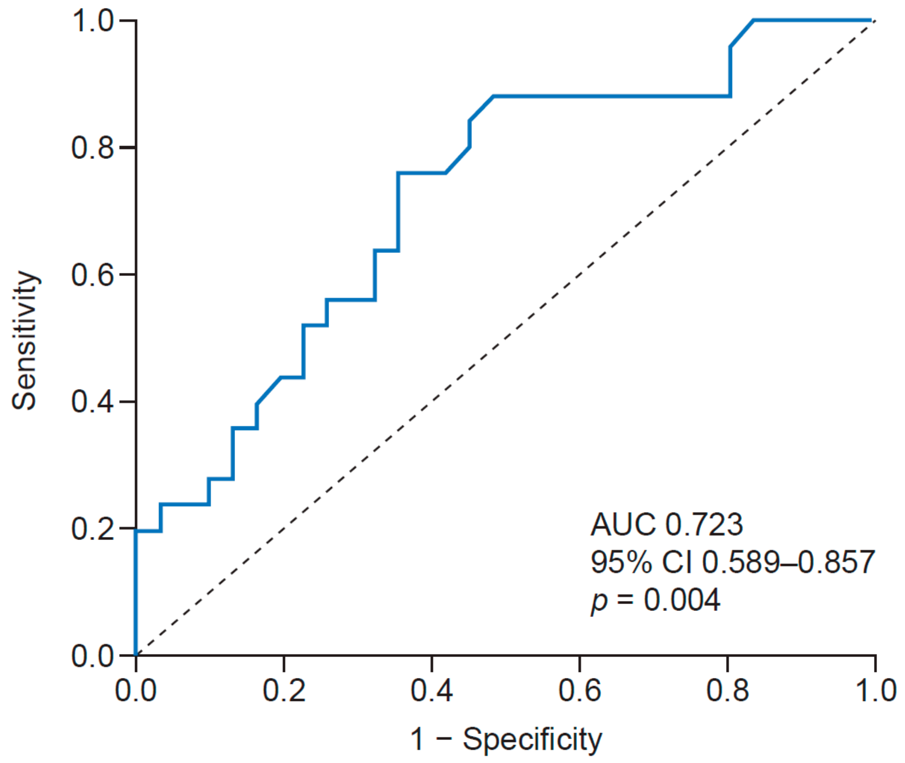

Figure S6 Receiver operating characteristic curves for the prediction of CLAD by ASW in early measurements (TP1). Diagonal segments are produced by ties. ASW, alveolar septal width; AUC, area under the curve; CI, confidence interval; CLAD, chronic lung allograft dysfunction; TP, timepoint.

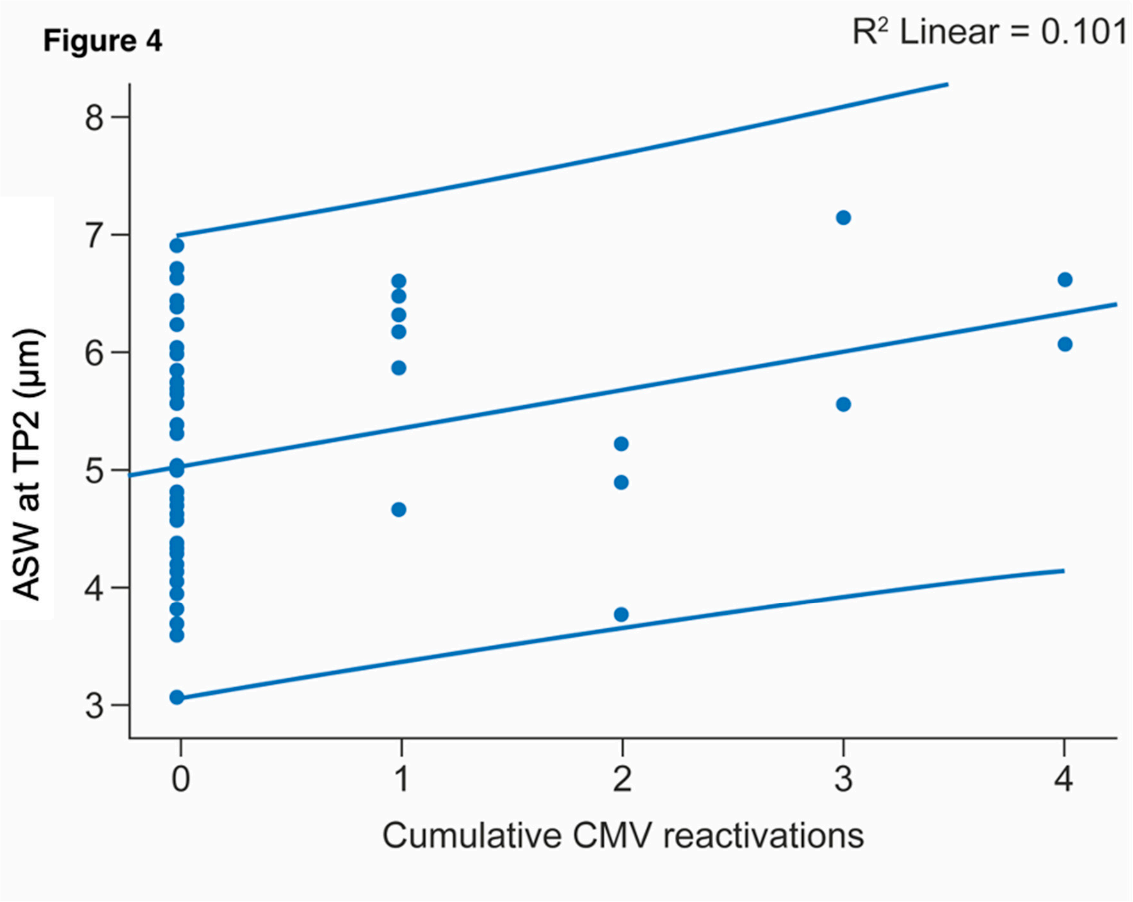

Figure S7 Scatter plot showing the association of ASW in late measurements (TP3) with the cumulative number of CMV reactivations. Error bars indicate the 95% confidence interval. ASW, alveolar septal width; CMV, cytomegalovirus; TP, timepoint.

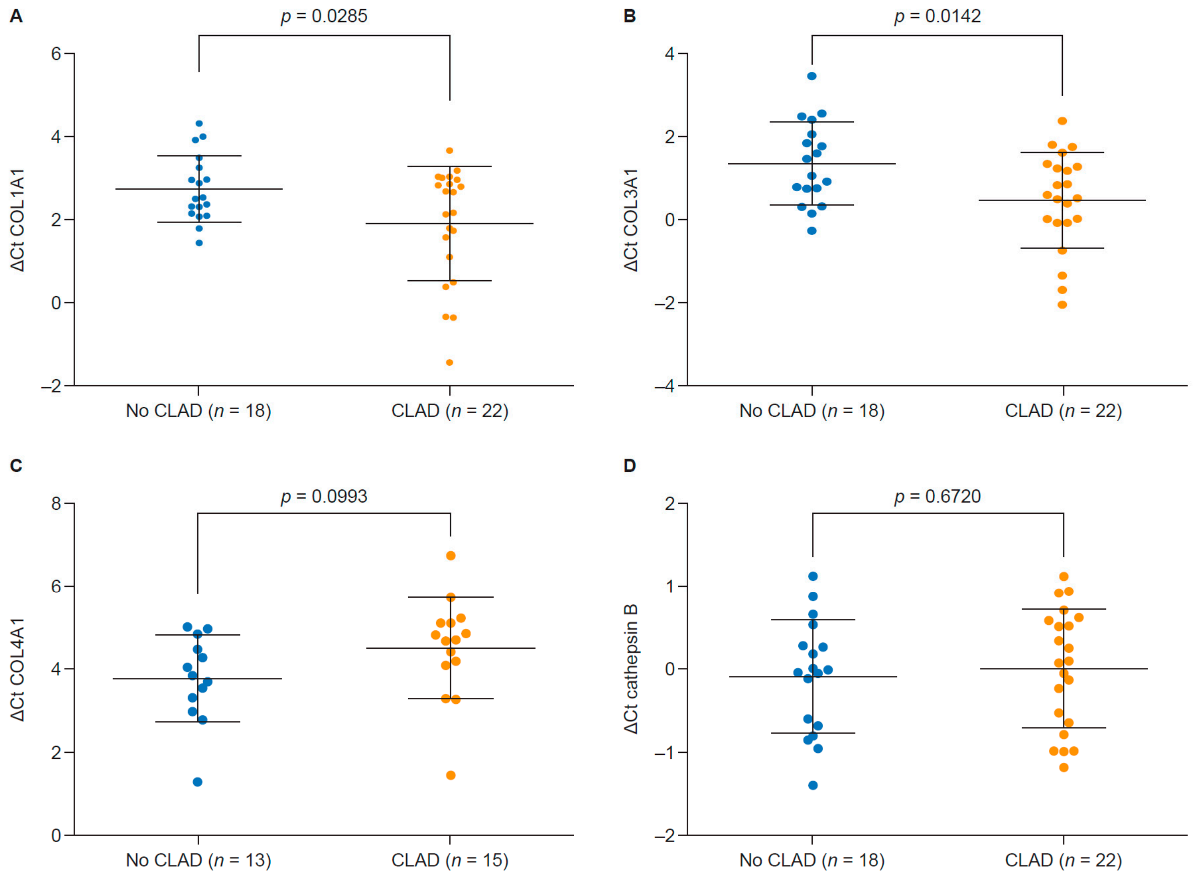

Figure S8 ΔC_t values of expression of COL1A1, COL3A1, COL4A1, and cathepsin B in patients who developed CLAD and those who did not. One ΔCt value was excluded as an outlier. Horizontal lines indicate the median, and whiskers the 95% confidence interval. CLAD, chronic lung allograft dysfunction; COL, collagen.

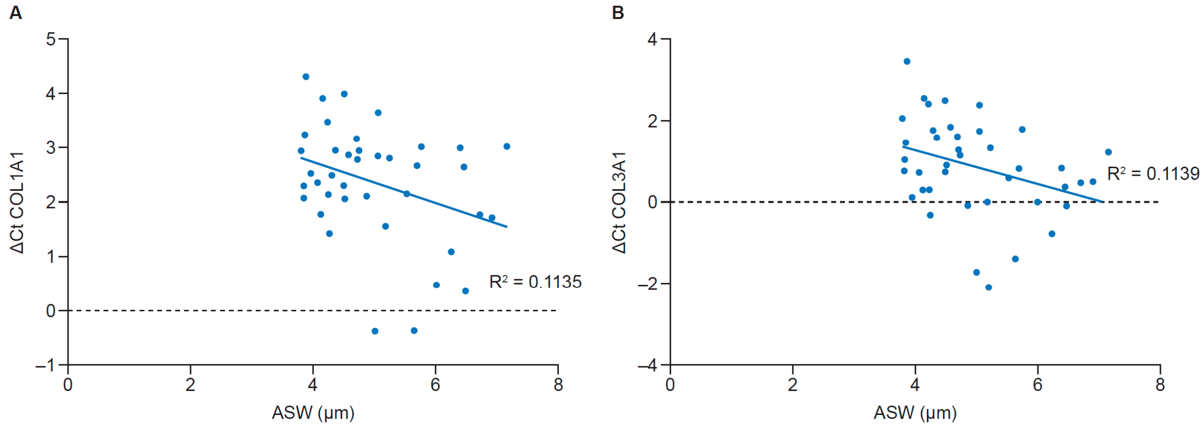

Figure S9 Manual measurements of ASW in correlation with ΔC_t values for expression of (A) COL1A1 ($p = 0.036$) and (B) COL3A1 ($p = 0.033$). Correlations of ASW with ΔC_t values for COL4A1 and cathepsin B expression were not significant (COL4A1: $R^2 = 0.197$, $p = 0.097$; cathepsin B: $R^2 = 0.035$, $p = 0.973$). One ΔCt value was excluded as an outlier. ASW, alveolar septal width; COL, collagen.

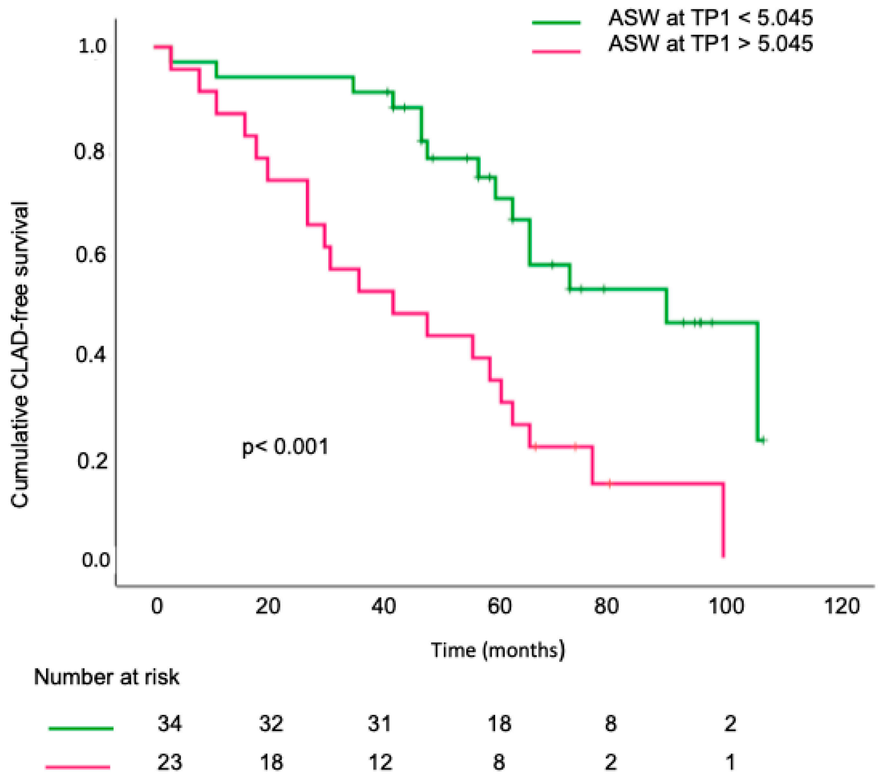

Figure S10 Kaplan–Meier CLAD – free survival curves for patients with ASW early after lung transplant (TP1) over and under 5.045 µm. ASW, alveolar septal width; TP, timepoint.

Supplemental Tables

Table S1 Definition of CLAD Phenotypes Adapted from Verleden et al.[4]

**CLAD phenotype**	**Obstructive (FEV_1/FVC < 0.7)**	**Restrictive (TLC < 90% of baseline[a])**	**Parenchymal/pleural opacities[b] in CT scans**
BOS	Yes	No	No
RAS	No	Yes	Yes
Mixed	Yes	Yes	Yes
Undefined	Yes	No	Yes
	Yes	Yes	No

BOS, bronchiolitis obliterans syndrome; CLAD, chronic lung allograft dysfunction; CT, computed tomography; FEV_1, forced expiratory volume in 1 second; FVC, forced vital capacity; RAS, restrictive allograft syndrome; TLC, total lung capacity.

[a]The mean of the two best measurements (taken >3 weeks apart) in the first 12 months post-LuTX define baseline.

[b]Opacities may be parenchymal opacities and/or pleural thickening as diagnosed in pulmonary fibrosis.

Table S2 Additional Donor and Recipient Characteristics and Peri- and Post-Operative Factors

	CLAD			p
	**No (*n* = 31)**	**Yes (*n* = 26)**	**All (*n* = 57)**	
**Donor characteristics**				
Height, cm	173 [165–184]	170 [166–180]	170 [165–180]	0.92
Weight, kg	77.1 ± 15.8	75.6 ± 11.1	76.5 ± 13.7	0.81
TLC, L	6.18 [5.1–7.62]	6.38 [5.25–7.3]	6.26 [5.14–7.3]	0.89
CRP, mg/L	136 [58–217]	122 [62–193]	125 [62–213]	0.94
Rhesus factor, *n* (%)				0.22
negative	9 (29)	4 (15)	13 (23)	
positive	22 (71)	22 (85)	44 (77)	
EBV serostatus, *n* (%)				0.13
Negative	0 (0)	2 (8)	2 (4)	
Positive	27 (87)	23 (88)	50 (88)	
Missing	4 (13)	1 (4)	5 (9)	
**Recipient characteristics**				
Height, cm	171.3 ± 9.5	170.6 ± 9.5	171.2 ± 9.5	1.0
Weight, kg	68.03 ± 12.37	73.87 ± 17.0	68.47 ± 14.98	0.19
EBV serostatus, *n* (%)				0.35

Negative	3 (11)	1 (4)	4 (7)	
Positive	25 (89)	22 (96)	47 (96)	
**Peri- and post-operative factors**				
Statin therapy before LuTX, *n* (%)				0.51
No	26 (84)	20 (77)	46 (81)	
Yes	5 (16)	6 (23)	11 (19)	
ECMO before LuTX, *n* (%)				0.71
No	29 (94)	23 (88)	52 (91)	
Yes	2 (6)	1 (4)	3 (5)	
Missing	0 (0)	2 (8)	2 (4)	
Duration of MAP <60 mm Hg, min	27 (12–65)	22 (12–65)	26(12–64)	0.73
Cumulative vasopressor dose, µg	2515 [1152–4045]	1975 [1276–3547]	2123 [1172–3833]	0.48
Number of FFP units transfused	0 [0–4]	0 [0–2]	0 [0–3]	0.68
Number of platelet units transfused	0 (0)	0 (0)	0 (0)	0.13
LOS in hospital, d	35 [21–65]	40 [22–65]	35 [21–60]	0.50

Cumulative EBV reactivation, *n* (%)[a]	0 (0)	0 (0)	0 (0)	0.75
Switch mycophenolate to everolimus, *n* (%)				0.37
Yes	13 (42)	14 (54)	27 (47)	
No	18 (58)	12 (46)	30 (53)	
Best $MMEF_{25–75}$, L/s	2.2 [1.8–3.7]	2.2 [1.4–3.4]	2.2 [1.5–3.6]	0.48
Best FEV_1, L	2.7 ± 0.7	2.6 ± 0.9	2.7 ± 0.8	0.47
Best TLC, L	5.5 ± 0.9	5.5 ± 1.2	5.5 ± 1.0	0.85
Best FVC, L	3.3 [3.0–4.2]	3.2 [2.6–3.8]	3.2 [2.8–4.0]	0.38

CLAD, chronic lung allograft dysfunction; CRP, C-reactive protein; EBV, Eppstein–Barr virus; ECMO, extracorporeal membrane oxygenation; FEV_1, forced expiratory volume in 1 second; FFP, fresh frozen plasma; FVC, forced vital capacity; LuTX, lung transplantation; MAP, mean arterial pressure; $MMEF_{25–75}$, maximal mid-expiratory flow at 25–75% of FVC; TLC, total lung capacity.

[a]From LuTX to timepoint 3.

Data are presented as mean ± SD, median (Q1–Q3) or *n* (%).

Table S3 Associations with Mortality in Univariate and Multivariate Regression Models

	**Univariate**		**Multivariate**	
	**HR (95% CI)**	***p***	**HR (95% CI)**	***p***
Age	1.003 (0.979–1.028)	0.810		
Sex	0.407 (0.162–1.024)	0.056		
Slight smoking	1.014 (0.288–4.519)	0.985		
Medium smoking	0.813 (0.105–6.314)	0.843		
Strong smoking	0.315 (0.041–2.418)	0.267		
Days on ventilation	0.930 (0.795–1.088)	0.362		
PO_2 100%	1.007 (1.000–1.014)	0.044	1.010 (1.003–1.017)	0.003
EBV serostatus	0.785 (0.101–6.082)	0.816		
CMV serostatus	1.381 (0.570–3.346)	0.475		

Age	0.988 (0.958–1.020)	0.462	1.002 (0.968–1.038)	0.896
Sex	0.514 (0.196–1.347)	0.176	0.613 (0.201–1.868)	0.389
Body mass index	0.959 (0.847-1.085)	0.509		
Immunosuppression	0.045 (0.00–1747.677)	0.566		
COPD/emphysema	5.358 (0.715–40.164)	0.102		
Fibrosis – IPF	1.465 (0.294–7.298)	0.641		
Fibrosis – other	3.354 (0.680–16.551)	0.137		
Cystic fibrosis	2.492 (0.412–15.099)	0.320		
EBV mismatch D/R	0.817 (0.186–3.596)	0.789		
CMV mismatch D/R	2.340 (0.385–14.239)	0.260		
Last LAS[a]	1.010 (0.986–1.036)	0.416		
ECMO before LuTX	2.081 (0.478–9.070)	0.329		

ECMO during surgery	2.804 (1.121–7.014)	0.028	7.71 (2.339–25.421)	<0.001
Incision–suture time	1.003 (0.997–1.009)	0.387		
Ischemic time	1.001 (0.996–1.007)	0.367		
Duration of MAP <60 mm Hg	1.004 (1.00–1.015)	0.494		
Cumulative vasopressor dose	1.00 (1.000–1.000)	0.142		
No. of RBC units transfused	1.122 (0.993–1.267)	0.064		
Post-LuTX time on ventilator[b]	1.002 (1.001–1.003)	0.004	1.000 (0.999–1.002)	0.620
LOS in ICU[b]	1.024 (1.006–1.042)	0.009		
Single lung vs double lung	0.611 (0.140–2.685)	0.511		
Cumulative no. of acute rejections	1.246 (0.760–2.041)	0.383		
Cumulative no. of EBV infections	1.262 (0.754–2.112)	0.375		
Cumulative no. of CMV infections	1.616 (1.128–2.315)	0.009	1.800 (1.133–2.858)	0.013

ASW at TP1	1.636 (1.081–2.476)	0.020[c]		
ASW at TP2	1.823 (1.132–2.935)	0.013	1.885 (1.086–3.269)	0.024

ASW alveolar septal width; CI, confidence interval; CMV, cytomegalovirus; COPD, chronic obstructive pulmonary disease; D/R, donor/recipient; EBV, Eppstein–Barr virus; ECMO, extracorporeal membrane oxygenation; HR, hazard ratio; ICU, intensive care unit; IPF, idiopathic pulmonary fibrosis; LAS, lung allocation score; LOS, length of stay; LuTX, lung transplantation; MAP, mean arterial pressure; PO_2 100%, arterial oxygen pressure in mm Hg under ventilation with 100% oxygen; RBC, red blood cell; TP, timepoint.

[a]LAS is a numerical value used by the United Network for Organ Sharing to assign relative priority for distributing donated lungs for transplantation and ranges from 0 to 1000, with a higher score indicating greater priority.

[b]Owing to multicollinearity (R = 0.787) between LOS in ICU and post-LuTX time on ventilator, only the latter was included in the multivariate model as the better defined and more precise parameter.

[c]ASW at TP1 could not be confirmed as an independent risk factor for mortality in multivariate analysis.

All available covariates were included in the univariate analysis, while only variables with a significance level of below 0.05 and recipient age and sex were used for the multiple regression models.

Table S4 Manual short protocol measurements and KI analysis at TP1

		Mean non-CLAD	Mean CLAD	p
Field A	Manual ASW [µm]	4.71 (0.64)	7.38 (1.57)	<0.001
	Sum (Area) [µm²]	107630.66 (31316.94)	138731.50 (33913.74)	0.047
	Area Fraction ROI [%]	40.54 (11.80)	52.26 (12.77)	0.047
Field B	Manual ASW [µm]	4.49 (0.87)	7.12 (1.18)	0.002
	Sum (Area) [µm²]	128249.84 (33658.60)	151150.49 (30294.67)	0.127
	Area Fraction ROI [%]	48.31 (12.68)	56.93 (11.41)	0.127
Fields A/B	Manual ASW [µm]	4.60 (0.73)	7.25 (1.28)	<0.001
	Sum (Area) [µm²]	117940.25 (27256.40)	144941.00 (24117.37)	0.031
	Area Fraction ROI [%]	44.42 (10.27)	54.59 (9.08)	0.031

ASW, alveolar septal width; CLAD, chronic lung allograft dysfunction; ROI, region of interest.

Data are presented as mean ± SD

Supplemental references

1. Richtlinie gemäß § 16 Abs. 1 S. 1 Nrn. 2 u. 5 TPG für die Wartelistenführung und Organvermittlung zur Lungentransplantation. Dtsch Arztebl Int 2017;114(42): A-1948 / B-1648 / C-1614.

2. Weill D, Benden C, Corris PA, et al. A consensus document for the selection of lung transplant candidates: 2014--an update from the Pulmonary Transplantation Council of the International Society for Heart and Lung Transplantation. J Heart Lung Transplant 2015;34:1-15.

3. Schneck E, Askevold I, Rath R, et al. Chronic Lung Allograft Dysfunction Is Associated with Increased Levels of Cell-Free Mitochondrial DNA in Bronchoalveolar Lavage Fluid of Lung Transplant Recipients. J Clin Med 2022;11.

4. Verleden GM, Glanville AR, Lease ED, et al. Chronic lung allograft dysfunction: Definition, diagnostic criteria, and approaches to treatment-A consensus report from the Pulmonary Council of the ISHLT. J Heart Lung Transplant 2019;38:493-503.
